# (7aR*,12bS*)-8,12b-Dihydro-7a*H*-indeno­[1′,2′:5,6][1,4]selenazino[2,3,4-*ij*]quinolin-13-ium hydrogen sulfate

**DOI:** 10.1107/S1600536811047167

**Published:** 2011-11-12

**Authors:** Gunay Z. Mammadova, Zhanna V. Matsulevich, Galina N. Borisova, Alexander V. Borisov, Victor N. Khrustalev

**Affiliations:** a Baku State University, Z. Khalilov St. 23, Baku AZ-1148, Azerbaijan; b R.E. Alekseev Nizhny Novgorod State Technical University, 24 Minin St., Nizhny Novgorod, 603950 Russian Federation; c X-Ray Structural Centre, A.N. Nesmeyanov Institute of Organoelement Compounds, Russian Academy of Sciences, 28 Vavilov St., B-0334 Moscow, 119991 Russian Federation

## Abstract

In the title compound, C_18_H_14_NSe^+^·HSO_4_^−^, the cyclo­pentene ring in the cation has an envelope conformation while the central six-membered 1,4-selenazine ring adopts a sofa conformation. The dihedral angle between the planes of the terminal benzene rings is 68.08 (11)°. In the crystal, the anions form chains along the *c* axis through O—H⋯O hydrogen bonds. Weak C—H⋯O and C—H⋯π hydrogen bonds, as well as attractive Se⋯Se [3.5608 (8) Å] inter­actions, further consolidate the crystal structure.

## Related literature

For the synthesis and biological properties of selenium- and nitro­gen-containing heterocycles, see: Mugesh *et al.* (2001 ▶); Koketsu & Ishihara (2003 ▶); Nogueira *et al.* (2004 ▶); Bhabak & Mugesh (2007 ▶); Mlochowski & Giurg (2009 ▶); Back (2009 ▶); Mukherjee *et al.* (2010 ▶). For related compounds, see: Wright (2001 ▶); Garud *et al.* (2007 ▶); Sommen *et al.* (2007 ▶); Borisov *et al.* (2011 ▶).
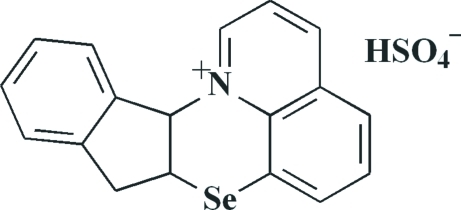


## Experimental

### 

#### Crystal data

C_18_H_14_NSe^+^·HSO_4_^−^
*M* *_r_* = 420.34
Monoclinic, 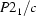

*a* = 11.1355 (11) Å
*b* = 19.5653 (19) Å
*c* = 7.9609 (8) Å
β = 107.005 (2)°
*V* = 1658.6 (3) Å^3^
*Z* = 4
Mo *K*α radiation
μ = 2.41 mm^−1^
*T* = 120 K
0.20 × 0.02 × 0.02 mm

#### Data collection

Bruker SMART 1K CCD diffractometer
Absorption correction: multi-scan (*SADABS*; Sheldrick, 1998 ▶) *T*_min_ = 0.644, *T*_max_ = 0.953
14396 measured reflections
4007 independent reflections
2902 reflections with *I* > 2σ(*I*)
*R*_int_ = 0.054

#### Refinement

*R*[*F* ^2^ > 2σ(*F* ^2^)] = 0.044
*wR*(*F* ^2^) = 0.114
*S* = 1.00
4007 reflections
226 parameters
H-atom parameters constrained
Δρ_max_ = 1.00 e Å^−3^
Δρ_min_ = −0.51 e Å^−3^

### 

Data collection: *SMART* (Bruker, 1998 ▶); cell refinement: *SAINT* (Bruker, 1998 ▶); data reduction: *SAINT*; program(s) used to solve structure: *SHELXTL* (Sheldrick, 2008 ▶); program(s) used to refine structure: *SHELXTL*; molecular graphics: *SHELXTL*; software used to prepare material for publication: *SHELXTL*.

## Supplementary Material

Crystal structure: contains datablock(s) global, I. DOI: 10.1107/S1600536811047167/rk2313sup1.cif

Structure factors: contains datablock(s) I. DOI: 10.1107/S1600536811047167/rk2313Isup2.hkl

Additional supplementary materials:  crystallographic information; 3D view; checkCIF report

## Figures and Tables

**Table 1:** Hydrogen-bond geometry (Å, °) *Cg* is the centroid of the C3*A*/C4–C6/C6*A*/C13*A* ring.

*D*—H⋯*A*	*D*—H	H⋯*A*	*D*⋯*A*	*D*—H⋯*A*
O4—H4*O*⋯O3^i^	0.96	1.59	2.548 (3)	171
C1—H1⋯O1	0.95	2.19	3.114 (4)	165
C3—H3⋯O2^ii^	0.95	2.28	3.154 (4)	153
C4—H4⋯O2^ii^	0.95	2.49	3.308 (4)	144
C5—H5⋯O4^iii^	0.95	2.55	3.367 (4)	145
C7*A*—H7*A*⋯O2^iv^	1.00	2.49	3.367 (4)	146
C12*B*—H12*B*⋯O1^i^	1.00	2.42	3.244 (4)	139
C12*B*—H12*B*⋯O3^i^	1.00	2.57	3.355 (4)	135
C11—H11⋯*Cg*^v^	0.95	2.78	3.679 (4)	159

Symmetry codes: (i) 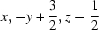; (ii) 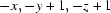; (iii) 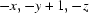; (iv) 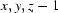; (v) 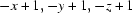.

**Fig. 1.**
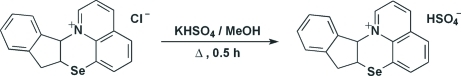
Reaction of 8,12b-dihydro-7aH-indeno[1',2':5,6][1,4]selenazino[2,3,4- ij]quinolin-13-ium chloride with potassium hydrosulfate.

**Fig. 2.**
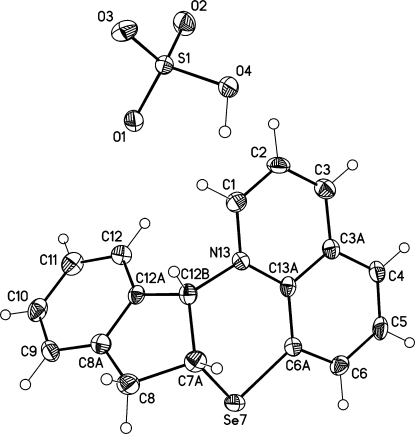
Molecular structure of I. Displacement ellipsoids are shown at the 50% probability level.

**Fig. 3.**
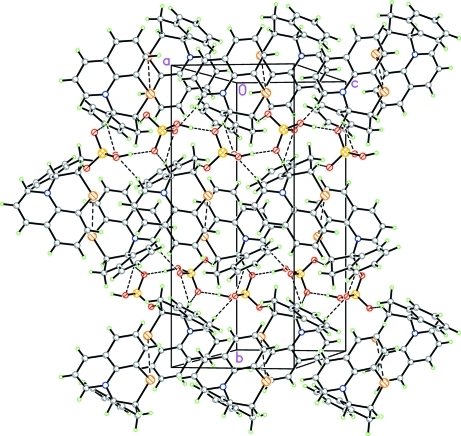
Crystal packing of I demonstrating the anionic chains along the c axis. Dashed lines indicate the intermolecular hydrogen bonding and attractive Se···Se interactions.

## supplementary crystallographic information

### Comment

In the last years, the selenium- and nitrogen-containing heterocycles have attracted considerable attention owing to the variety of their pharmacological properties (Mugesh *et al.*, 2001; Wright, 2001; Koketsu & Ishihara, 2003; Nogueira *et al.*, 2004; Bhabak & Mugesh, 2007; Garud *et al.*, 2007; Sommen *et al.*, 2007; Back, 2009; Mlochowski & Giurg, 2009; Mukherjee *et al.*, 2010). This article describes the structure of 8,12*b*-dihydro-7*aH*-indeno[1',2':5,6][1,4]selenazino[2,3,4- *ij*]quinolin-13-ium hydrosulfate, which was obtained by a reaction of 8,12*b*-dihydro-7a*H*-indeno[1',2':5,6][1,4]selenazino[2,3,4- *ij*]quinolin-13-ium chloride (Borisov *et al.*, 2011) with potassium hydrosulfate (Fig. 1).

The title compound of I, [C_18_H_14_NSe][HSO_4_], is a salt consisting of indeno[1',2':5,6][1,4]selenazino[2,3,4-*ij*]quinolin-13-ium cation and hydrosulfate anion. The cation of I comprises a fused pentacyclic system containing one five-membered ring (cyclopentene) and four six-membered rings (two benzene, 3,6-dihydro-1,4-selenazine and pyridine) (Fig. 2). The cyclopentene ring has the usual *envelope* conformation (the C7A carbon atom is out of the plane through the other atoms of the ring by 0.549 (5)Å)), and the central six-membered 1,4-selenazine ring adopts a *sofa* conformation (the C7A carbon atom is out of the plane through the other atoms of the ring by 0.677 (4)Å). The dihedral angle between the planes of the terminal benzene rings is 68.08 (11)°.

In the crystal, anions of I form chains along the *c* axis through the intermolecular O4—H4O···O3^i^ hydrogen bonding interactions (Table 1, Fig. 3). Weak intermolecular C—H···O (Table 1) and C11—H11···π (C3A^v^–C4^v^) (the H11···C3A^v^ and H11···C4^v^ distances are 2.79Å and 2.86Å, respectively) hydrogen bonds as well as attractive Se···Se^vi^ (3.5608 (8)Å) interactions consolidate further the three-dimensional crystal packing (Fig. 3). Symmetry codes: (i) *x*, -*y*+3/2, *z*-1/2; (v) -*x*+1, -*y*+1, -*z*; (vi) -*x*+1, -*y*+1, -*z*+1.

The cation of I possesses two asymmetric centers at the C7A and C12B carbon atoms and can have potentially four diastereomers. The crystal of I is racemic and consists of enantiomeric pairs with the following relative configuration of the centers: *rac*-7a*R**,12*bS**.

### Experimental

A mixture of 8,12*b*-dihydro-7a*H*-indeno[1',2':5,6][1,4]selenazino[2,3,4- *ij*]quinolin-13-ium chloride (0.147 g, 0.4 mmol) with KHSO_4_ (0.057 g, 0.42 mmol) in CH_3_OH (20 ml) was refluxed for 0.5 h to dissolve the starting materials. After that the reaction mixture was concentrated in *vacuo*. Then CH_2_Cl_2_ (20 ml) was added to the solid to give precipitate of KCl which was separated by filtration. The filtrate was concentrated in *vacuo*. The solid was recrystallized from CH_2_Cl_2_ to give I as orange needles. Yield is 89%. M.p. = 502–503 K. IR (KBr), ν (cm^-1^): 3481, 2987, 1608, 1552, 1485, 1440, 1294, 1172, 1134, 796, 721, 468, 419. ^1^H NMR (DMSO-d_6_, 300 MHz, 302 K): δ = 9.72 (dd, 1H, H1, J = 6.0, J = 1.3), 9.45 (dd, 1H, H3, J = 8.4, J = 1.4), 8.37 (dd, 1H, H2, J = 8.3, J = 5.8), 8.28 (dd, 1H, H4, J = 8.1, J = 1.3), 8.23 (dd, 1H, H6, J = 7.5, J = 1.3), 7.83 (t, 1H, H5, J = 7.8), 7.52 (d, 1H, H9, J = 7.5), 7.31 (t, 1H, H10, J = 7.5), 7.14 (t, 1H, H11, J = 7.5), 6.89 (d, 1H, H12*b*, J = 4.7), 6.60 (d, 1H, H12, J = 7.6), 4.87 (t, 1H, H7a, J = 4.7), 3.59 (dd, 1H, H8, J = 16.8, J = 4.7), 3.25 (d, H8, J = 16.8). Anal. Calcd. for C_18_H_15_NO_4_SSe: C, 51.43; H, 3.59; N, 3.33. Found: C, 51.34; H, 3.52; N, 3.29.

### Refinement

The hydroxyl hydrogen atom was localized in the difference-Fourier map and included in the refinement with fixed positional and isotropic displacement parameters [*U*_iso_(H) = 1.5*U*_eq_(O)]. The other hydrogen atoms were placed in calculated positions with C—H = 0.95–1.00Å and refined in the riding model with fixed isotropic displacement parameters [*U*_iso_(H) = 1.2*U*_eq_(C)].

### Crystal data

**Table:**

C_18_H_14_NSe^+^·HSO_4_^−^	*F*(000) = 848
*M**_r_* = 420.34	*D*_x_ = 1.683 Mg m^−^^3^
Monoclinic, *P*2_1_/*c*	Melting point = 502–503 K
Hall symbol: -P 2ybc	Mo *K*α radiation, λ = 0.71073 Å
*a* = 11.1355 (11) Å	Cell parameters from 2341 reflections
*b* = 19.5653 (19) Å	θ = 2.2–24.8°
*c* = 7.9609 (8) Å	µ = 2.41 mm^−^^1^
β = 107.005 (2)°	*T* = 120 K
*V* = 1658.6 (3) Å^3^	Needle, orange
*Z* = 4	0.20 × 0.02 × 0.02 mm

### Data collection

**Table:**

Bruker SMART 1K CCD diffractometer	4007 independent reflections
Radiation source: fine-focus sealed tube	2902 reflections with *I* > 2σ(*I*)
graphite	*R*_int_ = 0.054
φ and ω scans	θ_max_ = 28.0°, θ_min_ = 1.9°
Absorption correction: multi-scan (*SADABS*; Sheldrick, 1998)	*h* = −14→14
*T*_min_ = 0.644, *T*_max_ = 0.953	*k* = −24→25
14396 measured reflections	*l* = −10→10

### Refinement

**Table:**

Refinement on *F*^2^	Primary atom site location: structure-invariant direct methods
Least-squares matrix: full	Secondary atom site location: difference Fourier map
*R*[*F*^2^ > 2σ(*F*^2^)] = 0.044	Hydrogen site location: difference Fourier map
*wR*(*F*^2^) = 0.114	H-atom parameters constrained
*S* = 1.00	*w* = 1/[σ^2^(*F*_o_^2^) + (0.054*P*)^2^ + 2.36*P*] where *P* = (*F*_o_^2^ + 2*F*_c_^2^)/3
4007 reflections	(Δ/σ)_max_ = 0.001
226 parameters	Δρ_max_ = 1.00 e Å^−^^3^
0 restraints	Δρ_min_ = −0.51 e Å^−^^3^

### Special details

**Table:**

Geometry. All s.u.'s (except the s.u. in the dihedral angle between two l.s. planes) are estimated using the full covariance matrix. The cell s.u.'s are taken into account individually in the estimation of s.u.'s in distances, angles and torsion angles; correlations between s.u.'s in cell parameters are only used when they are defined by crystal symmetry. An approximate (isotropic) treatment of cell s.u.'s is used for estimating s.u.'s involving l.s. planes.
Refinement. Refinement of *F*^2^ against ALL reflections. The weighted *R*-factor *wR* and goodness of fit *S* are based on *F*^2^, conventional *R*-factors *R* are based on *F*, with *F* set to zero for negative *F*^2^. The threshold expression of *F*^2^ > σ(*F*^2^) is used only for calculating *R*-factors(gt) *etc*. and is not relevant to the choice of reflections for refinement. *R*-factors based on *F*^2^ are statistically about twice as large as those based on *F*, and *R*-factors based on ALL data will be even larger.

### Fractional atomic coordinates and isotropic or equivalent isotropic displacement parameters (Å^2^)

**Table:**

	*x*	*y*	*z*	*U*_iso_*/*U*_eq_	
C1	0.1870 (3)	0.58708 (18)	0.3376 (5)	0.0216 (7)	
H1	0.2013	0.6277	0.4059	0.026*	
C2	0.1119 (3)	0.53643 (18)	0.3759 (5)	0.0242 (8)	
H2	0.0772	0.5420	0.4708	0.029*	
C3	0.0884 (3)	0.47893 (19)	0.2765 (5)	0.0236 (7)	
H3	0.0365	0.4441	0.3010	0.028*	
C3A	0.1413 (3)	0.47090 (17)	0.1365 (4)	0.0183 (7)	
C4	0.1189 (3)	0.40994 (19)	0.0350 (5)	0.0227 (7)	
H4	0.0648	0.3756	0.0564	0.027*	
C5	0.1763 (3)	0.40145 (19)	−0.0938 (5)	0.0246 (8)	
H5	0.1622	0.3608	−0.1621	0.030*	
C6	0.2556 (3)	0.45222 (19)	−0.1259 (4)	0.0224 (7)	
H6	0.2938	0.4452	−0.2167	0.027*	
C6A	0.2801 (3)	0.51193 (17)	−0.0303 (4)	0.0192 (7)	
Se7	0.40049 (3)	0.571862 (19)	−0.07394 (4)	0.02345 (12)	
C7A	0.3453 (3)	0.65434 (18)	0.0190 (4)	0.0218 (7)	
H7A	0.2719	0.6763	−0.0679	0.026*	
C8	0.4599 (3)	0.70229 (18)	0.0743 (4)	0.0242 (8)	
H8A	0.5125	0.6978	−0.0061	0.029*	
H8B	0.4334	0.7505	0.0757	0.029*	
C8A	0.5298 (3)	0.67800 (17)	0.2574 (4)	0.0208 (7)	
C9	0.6519 (3)	0.69027 (18)	0.3579 (5)	0.0237 (7)	
H9	0.7065	0.7171	0.3129	0.028*	
C10	0.6934 (3)	0.6625 (2)	0.5264 (5)	0.0274 (8)	
H10	0.7772	0.6704	0.5967	0.033*	
C11	0.6130 (3)	0.6235 (2)	0.5921 (5)	0.0274 (8)	
H11	0.6426	0.6048	0.7070	0.033*	
C12	0.4901 (3)	0.61157 (18)	0.4919 (4)	0.0215 (7)	
H12	0.4349	0.5851	0.5369	0.026*	
C12A	0.4499 (3)	0.63916 (16)	0.3249 (4)	0.0164 (6)	
C12B	0.3200 (3)	0.64029 (17)	0.1943 (4)	0.0182 (7)	
H12B	0.2770	0.6818	0.2221	0.022*	
N13	0.2398 (2)	0.58057 (13)	0.2079 (3)	0.0155 (5)	
C13A	0.2219 (3)	0.52230 (17)	0.1038 (4)	0.0165 (6)	
S1	0.07534 (8)	0.71231 (4)	0.61913 (10)	0.01767 (18)	
O1	0.1979 (2)	0.70926 (12)	0.5924 (3)	0.0217 (5)	
O2	0.0535 (2)	0.66144 (13)	0.7377 (3)	0.0268 (6)	
O3	0.0469 (2)	0.78149 (12)	0.6674 (3)	0.0232 (5)	
O4	−0.0231 (2)	0.69555 (14)	0.4394 (3)	0.0290 (6)	
H4O	0.0104	0.7065	0.3440	0.043*	

### Atomic displacement parameters (Å^2^)

**Table:**

	*U*^11^	*U*^22^	*U*^33^	*U*^12^	*U*^13^	*U*^23^
C1	0.0230 (17)	0.0187 (18)	0.0244 (17)	0.0016 (14)	0.0093 (14)	−0.0044 (13)
C2	0.0268 (18)	0.025 (2)	0.0273 (18)	−0.0002 (15)	0.0177 (15)	−0.0031 (14)
C3	0.0234 (17)	0.0207 (18)	0.0289 (18)	−0.0007 (14)	0.0111 (15)	0.0006 (14)
C3A	0.0149 (15)	0.0174 (17)	0.0213 (16)	0.0016 (13)	0.0034 (13)	−0.0001 (13)
C4	0.0169 (16)	0.0240 (19)	0.0252 (17)	−0.0037 (14)	0.0028 (14)	−0.0028 (14)
C5	0.0223 (18)	0.0213 (18)	0.0274 (18)	−0.0050 (14)	0.0028 (15)	−0.0100 (14)
C6	0.0197 (17)	0.0265 (19)	0.0206 (16)	0.0021 (15)	0.0052 (14)	−0.0053 (14)
C6A	0.0184 (16)	0.0206 (18)	0.0184 (15)	−0.0026 (13)	0.0049 (13)	−0.0009 (13)
Se7	0.0276 (2)	0.0244 (2)	0.02247 (19)	−0.00505 (15)	0.01375 (14)	−0.00375 (14)
C7A	0.0265 (18)	0.0193 (18)	0.0191 (16)	0.0015 (14)	0.0059 (14)	0.0041 (13)
C8	0.032 (2)	0.0197 (18)	0.0226 (17)	−0.0019 (15)	0.0101 (15)	0.0031 (14)
C8A	0.0270 (18)	0.0117 (16)	0.0262 (17)	0.0012 (14)	0.0119 (15)	−0.0013 (13)
C9	0.0201 (17)	0.0204 (18)	0.0335 (19)	−0.0041 (14)	0.0122 (15)	−0.0062 (15)
C10	0.0180 (17)	0.034 (2)	0.0269 (18)	0.0022 (15)	0.0015 (14)	−0.0095 (16)
C11	0.0248 (19)	0.029 (2)	0.0254 (18)	0.0051 (16)	0.0026 (15)	0.0003 (15)
C12	0.0204 (17)	0.0222 (18)	0.0214 (16)	0.0024 (14)	0.0055 (13)	0.0022 (14)
C12A	0.0169 (15)	0.0141 (16)	0.0179 (15)	0.0012 (13)	0.0046 (12)	−0.0031 (12)
C12B	0.0186 (16)	0.0150 (16)	0.0195 (16)	−0.0001 (13)	0.0034 (13)	0.0009 (13)
N13	0.0144 (13)	0.0135 (14)	0.0190 (13)	0.0012 (10)	0.0056 (10)	0.0001 (10)
C13A	0.0126 (14)	0.0155 (16)	0.0188 (15)	0.0018 (12)	0.0007 (12)	−0.0017 (12)
S1	0.0187 (4)	0.0188 (4)	0.0159 (4)	−0.0036 (3)	0.0057 (3)	−0.0017 (3)
O1	0.0203 (12)	0.0209 (13)	0.0244 (12)	−0.0030 (10)	0.0074 (10)	−0.0040 (10)
O2	0.0313 (14)	0.0236 (14)	0.0261 (13)	−0.0067 (11)	0.0095 (11)	0.0032 (10)
O3	0.0317 (14)	0.0212 (13)	0.0189 (11)	0.0032 (11)	0.0107 (10)	0.0012 (10)
O4	0.0227 (13)	0.0483 (17)	0.0163 (12)	−0.0130 (12)	0.0064 (10)	−0.0072 (11)

### Geometric parameters (Å, °)

**Table:**

C1—N13	1.336 (4)	C8—H8A	0.9900
C1—C2	1.386 (5)	C8—H8B	0.9900
C1—H1	0.9500	C8A—C9	1.382 (5)
C2—C3	1.356 (5)	C8A—C12A	1.391 (4)
C2—H2	0.9500	C9—C10	1.395 (5)
C3—C3A	1.414 (5)	C9—H9	0.9500
C3—H3	0.9500	C10—C11	1.390 (5)
C3A—C4	1.421 (5)	C10—H10	0.9500
C3A—C13A	1.422 (5)	C11—C12	1.388 (5)
C4—C5	1.368 (5)	C11—H11	0.9500
C4—H4	0.9500	C12—C12A	1.383 (5)
C5—C6	1.401 (5)	C12—H12	0.9500
C5—H5	0.9500	C12A—C12B	1.515 (4)
C6—C6A	1.377 (5)	C12B—N13	1.494 (4)
C6—H6	0.9500	C12B—H12B	1.0000
C6A—C13A	1.416 (4)	N13—C13A	1.389 (4)
C6A—Se7	1.888 (3)	S1—O2	1.441 (2)
Se7—C7A	1.948 (3)	S1—O1	1.443 (2)
C7A—C12B	1.527 (4)	S1—O3	1.467 (2)
C7A—C8	1.541 (5)	S1—O4	1.562 (2)
C7A—H7A	1.0000	O4—H4O	0.9632
C8—C8A	1.514 (5)		
			
N13—C1—C2	122.1 (3)	C9—C8A—C12A	120.2 (3)
N13—C1—H1	118.9	C9—C8A—C8	130.1 (3)
C2—C1—H1	118.9	C12A—C8A—C8	109.7 (3)
C3—C2—C1	119.4 (3)	C8A—C9—C10	118.8 (3)
C3—C2—H2	120.3	C8A—C9—H9	120.6
C1—C2—H2	120.3	C10—C9—H9	120.6
C2—C3—C3A	119.9 (3)	C11—C10—C9	120.5 (3)
C2—C3—H3	120.1	C11—C10—H10	119.8
C3A—C3—H3	120.1	C9—C10—H10	119.8
C3—C3A—C4	119.8 (3)	C12—C11—C10	120.8 (3)
C3—C3A—C13A	119.8 (3)	C12—C11—H11	119.6
C4—C3A—C13A	120.3 (3)	C10—C11—H11	119.6
C5—C4—C3A	119.0 (3)	C12A—C12—C11	118.3 (3)
C5—C4—H4	120.5	C12A—C12—H12	120.8
C3A—C4—H4	120.5	C11—C12—H12	120.8
C4—C5—C6	120.5 (3)	C12—C12A—C8A	121.4 (3)
C4—C5—H5	119.7	C12—C12A—C12B	129.9 (3)
C6—C5—H5	119.7	C8A—C12A—C12B	108.5 (3)
C6A—C6—C5	122.3 (3)	N13—C12B—C12A	114.2 (3)
C6A—C6—H6	118.8	N13—C12B—C7A	118.7 (3)
C5—C6—H6	118.8	C12A—C12B—C7A	103.6 (3)
C6—C6A—C13A	118.5 (3)	N13—C12B—H12B	106.5
C6—C6A—Se7	117.5 (3)	C12A—C12B—H12B	106.5
C13A—C6A—Se7	123.8 (2)	C7A—C12B—H12B	106.5
C6A—Se7—C7A	97.16 (15)	C1—N13—C13A	121.4 (3)
C12B—C7A—C8	102.0 (3)	C1—N13—C12B	112.9 (3)
C12B—C7A—Se7	111.2 (2)	C13A—N13—C12B	125.7 (3)
C8—C7A—Se7	106.6 (2)	N13—C13A—C6A	123.4 (3)
C12B—C7A—H7A	112.2	N13—C13A—C3A	117.3 (3)
C8—C7A—H7A	112.2	C6A—C13A—C3A	119.3 (3)
Se7—C7A—H7A	112.2	O2—S1—O1	114.60 (15)
C8A—C8—C7A	103.5 (3)	O2—S1—O3	112.03 (15)
C8A—C8—H8A	111.1	O1—S1—O3	111.29 (15)
C7A—C8—H8A	111.1	O2—S1—O4	104.34 (15)
C8A—C8—H8B	111.1	O1—S1—O4	107.28 (14)
C7A—C8—H8B	111.1	O3—S1—O4	106.62 (15)
H8A—C8—H8B	109.0	S1—O4—H4O	110.2
			
N13—C1—C2—C3	−1.6 (5)	C8—C8A—C12A—C12B	−4.2 (4)
C1—C2—C3—C3A	0.6 (5)	C12—C12A—C12B—N13	−30.0 (5)
C2—C3—C3A—C4	178.6 (3)	C8A—C12A—C12B—N13	155.7 (3)
C2—C3—C3A—C13A	1.9 (5)	C12—C12A—C12B—C7A	−160.7 (3)
C3—C3A—C4—C5	−176.5 (3)	C8A—C12A—C12B—C7A	25.1 (3)
C13A—C3A—C4—C5	0.2 (5)	C8—C7A—C12B—N13	−162.7 (3)
C3A—C4—C5—C6	−0.3 (5)	Se7—C7A—C12B—N13	−49.4 (4)
C4—C5—C6—C6A	0.4 (5)	C8—C7A—C12B—C12A	−34.8 (3)
C5—C6—C6A—C13A	−0.5 (5)	Se7—C7A—C12B—C12A	78.5 (3)
C5—C6—C6A—Se7	174.4 (3)	C2—C1—N13—C13A	0.1 (5)
C6—C6A—Se7—C7A	159.0 (3)	C2—C1—N13—C12B	−178.1 (3)
C13A—C6A—Se7—C7A	−26.4 (3)	C12A—C12B—N13—C1	80.4 (3)
C6A—Se7—C7A—C12B	44.9 (3)	C7A—C12B—N13—C1	−156.9 (3)
C6A—Se7—C7A—C8	155.2 (2)	C12A—C12B—N13—C13A	−97.7 (4)
C12B—C7A—C8—C8A	32.3 (3)	C7A—C12B—N13—C13A	25.0 (4)
Se7—C7A—C8—C8A	−84.4 (3)	C1—N13—C13A—C6A	−177.3 (3)
C7A—C8—C8A—C9	163.5 (3)	C12B—N13—C13A—C6A	0.6 (5)
C7A—C8—C8A—C12A	−18.1 (4)	C1—N13—C13A—C3A	2.3 (4)
C12A—C8A—C9—C10	0.6 (5)	C12B—N13—C13A—C3A	−179.7 (3)
C8—C8A—C9—C10	178.8 (3)	C6—C6A—C13A—N13	−179.9 (3)
C8A—C9—C10—C11	−0.3 (5)	Se7—C6A—C13A—N13	5.5 (5)
C9—C10—C11—C12	−0.2 (6)	C6—C6A—C13A—C3A	0.5 (5)
C10—C11—C12—C12A	0.3 (5)	Se7—C6A—C13A—C3A	−174.2 (2)
C11—C12—C12A—C8A	0.0 (5)	C3—C3A—C13A—N13	−3.3 (5)
C11—C12—C12A—C12B	−173.6 (3)	C4—C3A—C13A—N13	−179.9 (3)
C9—C8A—C12A—C12	−0.5 (5)	C3—C3A—C13A—C6A	176.4 (3)
C8—C8A—C12A—C12	−179.0 (3)	C4—C3A—C13A—C6A	−0.3 (5)
C9—C8A—C12A—C12B	174.4 (3)		

### Hydrogen-bond geometry (Å, °)

**Table:**

Cg is the centroid of the C3A/C4–C6/C6A/C13A ring.

**Table:**

*D*—H···*A*	*D*—H	H···*A*	*D*···*A*	*D*—H···*A*
O4—H4O···O3^i^	0.96	1.59	2.548 (3)	171
C1—H1···O1	0.95	2.19	3.114 (4)	165
C3—H3···O2^ii^	0.95	2.28	3.154 (4)	153
C4—H4···O2^ii^	0.95	2.49	3.308 (4)	144
C5—H5···O4^iii^	0.95	2.55	3.367 (4)	145
C7A—H7A···O2^iv^	1.00	2.49	3.367 (4)	146
C12B—H12B···O1^i^	1.00	2.42	3.244 (4)	139
C12B—H12B···O3^i^	1.00	2.57	3.355 (4)	135
C11—H11···Cg^v^	0.95	2.78	3.679 (4)	159

Symmetry codes: (i) *x*, −*y*+3/2, *z*−1/2; (ii) −*x*, −*y*+1, −*z*+1; (iii) −*x*, −*y*+1, −*z*; (iv) *x*, *y*, *z*−1; (v) −*x*+1, −*y*+1, −*z*+1.
